# The Polycomb Group Protein Ring1b/Rnf2 Is Specifically Required for Craniofacial Development

**DOI:** 10.1371/journal.pone.0073997

**Published:** 2013-09-11

**Authors:** Yme U. van der Velden, Liqin Wang, Laia Querol Cano, Anna-Pavlina G. Haramis

**Affiliations:** 1 Department of Molecular Genetics, Netherlands Cancer Institute, Amsterdam, The Netherlands; 2 Department of Medical Microbiology, Academic Medical Center, Amsterdam, The Netherlands; 3 Department of Molecular Carcinogenesis, Netherlands Cancer Institute, Amsterdam, The Netherlands; 4 Institute of Biology, Leiden University, Leiden, The Netherlands; Texas A&M University, United States of America

## Abstract

Polycomb group (PcG) genes are chromatin modifiers that mediate epigenetic silencing of target genes. PcG-mediated epigenetic silencing is implicated in embryonic development, stem cell plasticity, cell fate maintenance, cellular differentiation and cancer. However, analysis of the roles of PcG proteins in maintaining differentiation programs during vertebrate embryogenesis has been hampered due to the early embryonic lethality of several PcG knock-outs in the mouse. Here, we show that zebrafish Ring1b/Rnf2, the single E3 ubiquitin ligase in the Polycomb Repressive Complex 1, critically regulates the developmental program of craniofacial cell lineages. Zebrafish *ring1b* mutants display a severe craniofacial phenotype, which includes an almost complete absence of all cranial cartilage, bone and musculature. We show that Cranial Neural Crest (CNC)-derived cartilage precursors migrate correctly into the pharyngeal arches, but fail to differentiate into chondrocytes. This phenotype is specific for cartilage precursors, since other neural crest-derived cell lineages, including glia, neurons and chromatophores, are formed normally in *ring1b* mutants. Our results therefore reveal a critical and specific role for Ring1b in promoting the differentiation of cranial neural crest cells into chondrocytes. The molecular mechanisms underlying the pathogenesis of craniofacial abnormalities, which are among the most common genetic birth defects in humans, remain poorly understood. The zebrafish *ring1b* mutant provides a molecular model for investigating these mechanisms and may lead to the discovery of new treatments or preventions of craniofacial abnormalities.

## Introduction

Craniofacial abnormalities are amongst the most common genetic birth defects and result from defects in cranial neural crest patterning and morphogenesis. However, the molecular mechanisms regulating cranial neural crest differentiation are not completely understood. Most of the cartilage and bones of the skull in vertebrate embryos are formed by cranial neural crest cells (CNCs), a subpopulation of the neural crest (NC), a vertebrate-specific, ectoderm-derived, multipotent cell population that is induced at the beginning of neurulation at the border between epidermal and neural ectoderm [1], [2]. Fate-mapping studies in fish, amphibians, birds and mammals have shown that streams of NC cells from the hindbrain form most of the pharyngeal skeleton, while the more anterior NC cells form the brain-case or neurocranium [2], [3]. The NC gives rise not only to cartilage and bone, but also to neurons, glia, smooth muscle cells and chromatophores. Distinct cell types are formed according to their position along the anteroposterior axis. For instance, in teleosts, NC cells that give rise to chromatophores originate from trunk NC, whereas NC cells that will form craniofacial cartilage are located at the level of the mid/hindbrain [4], [5].

During cartilage development, the most anteriorly-located CNC-derived cartilage precursors will mainly form skeletal elements that encase the brain (neurocranium), whereas more posteriorly-located NC cells give rise to the ventral viscerocranium. Viscerocranial precursors migrate ventrally as three streams to populate the pharyngeal arches, embryonic structures that will form several tissues of the head-neck region in higher vertebrates. The mandibular and hyoid arches form the jaw and its supportive elements, respectively, whereas the five posterior (branchial) arches give rise to the cartilaginous elements of the gills.

Cranial bones are also derived from CNC cells and ossification occurs through two distinct processes: endochondral and dermal ossification. In endochondral ossification, a skeletal template comprising of replacement cartilage is formed first, which is then replaced by bone. In dermal ossification, bones form directly from osteogenic condensations. Both processes are essential for cranial bone development.

Skeletogenesis is a complex process guided by specific genetic and epigenetic programs. The Polycomb group (PcG) proteins are transcriptional repressors that mediate epigenetic silencing of target genes and thereby regulate differentiation programs. PcG proteins are key regulators of cell proliferation, cell-fate maintenance during embryonic development; and in adults, they are implicated in tissue homeostasis, cellular differentiation and cancer [6], [7]. Mechanisms underlying PcG-mediated gene silencing include the organization of higher-order chromatin structure, post-translational modifications on nucleosomes and interference with the transcription machinery [7], [8], [9]. The ubiquitin E3-ligase activity of RING-domain-containing proteins in Polycomb Repressive Complex 1 (PRC1) leads to the mono-ubiquitilation of histone H2A at lysine 119 (H2AK119), a histone modification that is associated with gene repression [10], [11].

The study of PcG proteins in maintaining differentiation programs during vertebrate embryonic development is hampered by the early lethality of mice defective for the core proteins of PRC2 and PRC1 (Ring1b). We have recently generated Ring1b-deficient zebrafish that survive early development (in contrast to mouse *ring1b* knock-outs [12]) and show surprisingly specific defects in tissue differentiation [13].

In this study we show that Ring1b-deficient zebrafish display a severe craniofacial phenotype, including an almost complete absence of all cranial cartilage, bone and musculature. We show that specification and initial migration of CNC-derived cartilage precursors into the pharyngeal arches is largely unaffected, however, cartilage differentiation is blocked. Moreover, the loss of Ring1b specifically affects CNC-derived cartilage precursors since other NC-derived lineages including neurons, glia and chromatophores are present in *ring1b* mutants. Finally, both endochondral and dermal ossification are disrupted due to loss of Ring1b function.

Our study highlights the importance of epigenetic control in cartilage differentiation and unravels a critical and specific role for PcG–mediated gene regulation in craniofacial development.

## Results and Discussion

### *Ring1b* Mutants Lack The Jaw Elements

The *ring1b/rnf2* mutants were generated by zinc finger nuclease (ZFN)-mediated targeted gene inactivation [14] and were previously described [13]. Briefly, two indel alleles were generated, which resulted in an open reading frame-shift that led to a premature stop codon. No Ring1b protein was produced and no ubiquitination of H2A was detected in the *ring1b* mutants [13], indicating that they are functional nulls.

**Figure 1:**
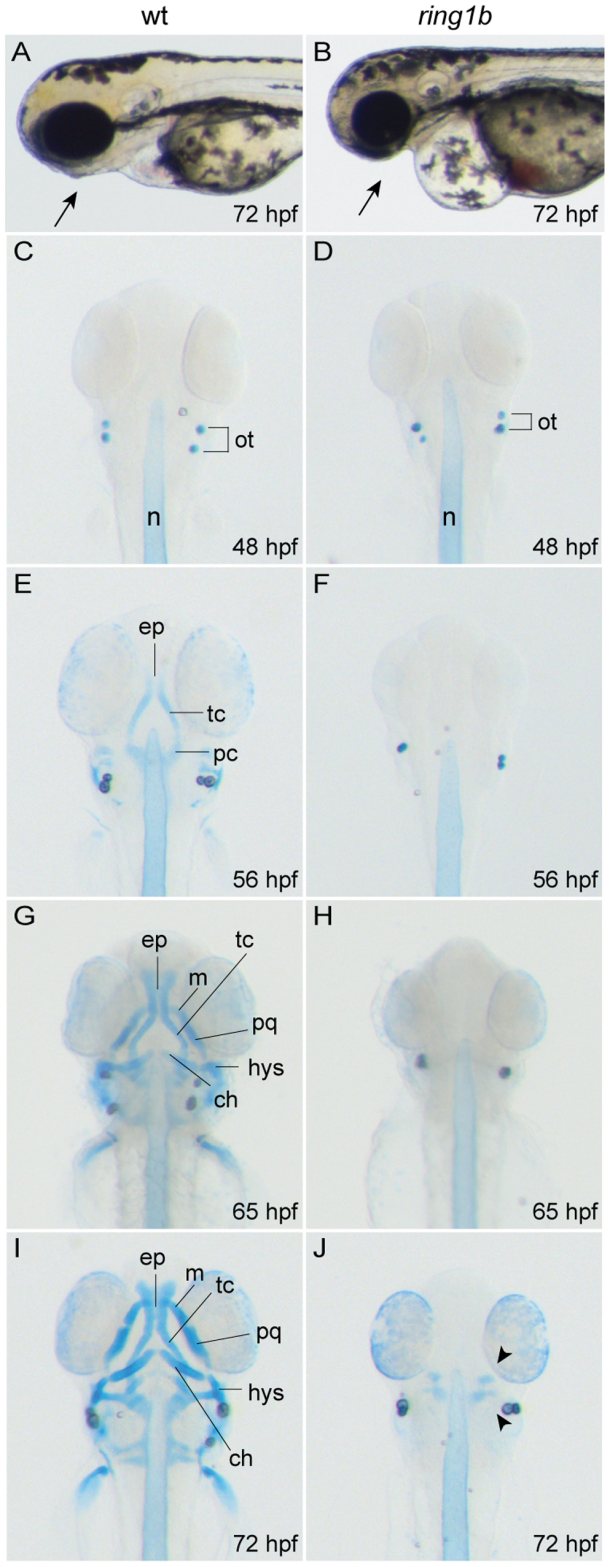
*Ring1b* mutants lack almost all head cartilage elements. Lateral view of WT and *ring1b* live embryos at 72 hpf (A, B). Alcian-Blue stained head cartilages of WT (C, E, G and H) and *ring1b* (D, F, H and J) mutants at the indicated developmental points, ventral views. The paired trabeculae have elongated and fused posteriorly in WT embryos at 56 hpf (E) and by 72 hpf the elaborate cartilagenous skeleton of the head has been established (I). In contrast, no cartilage is visible in *ring1b* mutants except for two minute cartilage deposits at 72 hpf *ring1b* mutants (J: arrowheads). ch: ceratohyal; ep: ethmoid plate; hys: hyosymplectic; m: Meckel’s cartilage; pc: parachordal, pq: palatoquadrate; tc: trabeculae.

Visible craniofacial defects in homozygous *ring1b* mutants were first observed at around 40 hours post fertilization (hpf), and consisted of a reduction in the amount of tissue at the level of the anterior pharyngeal arches. In contrast to WT embryos at 72 hpf (Fig. 1A), *ring1b* mutants showed a complete absence of tissue under the eye at the location of the jaw (Fig. 1B). Indeed, Alcian-Blue staining to visualize cartilage, revealed that almost all craniofacial and jaw cartilage elements were absent in *ring1b* mutants at 72 hpf. This dramatic phenotype led us to examine whether cartilage elements were formed at an earlier developmental stage and then deteriorated or whether any cartilage was formed at all. To address these issues, we stained WT and *ring1b* siblings at different developmental stages with Alcian blue.

In WT embryos at 56 hpf, the trabeculae cranii, a cartilage element of the prospective neurocranium, had fused posteriorly to the parachordals and the ethmoid plate starts to emerge anteriorly (Fig. 1E). At 65 hpf, the ethmoid plate had elongated in WT embryos and the first viscerocranial cartilage structures were also formed (Fig. 1G) as well as Meckel’s cartilage and the palatoquadrate (derived from cartilage precursors from the mandibular arch [15]. The ceratohyal and hyosymplectic cartilage elements, derived from the hyoid arch [15] had also differentiated in WT embryos (Fig. 1G). At 72 hpf, craniofacial cartilage structures had differentiated further and the elaborate cartilaginous skeleton of the head had been established (Fig. 1I). In contrast to WT embryos, craniofacial cartilages were absent in *ring1b* mutants at both 56 and 65 hpf (Fig. 1F, H). At 72 hpf, the entire head cartilage skeleton was missing with the exception of two cartilage deposits located at both sides of the anterior notochord (Fig. 1J). The cartilaginous pectoral fin girdle was also missing. Together, these results show that loss of Ring1b severely disrupts head skeleton formation.

### Cranial Muscle Development is Compromised in *ring1b* Mutants

Because both the viscerocranium and neurocranium are essential for the patterning of associated craniofacial musculature [16], we examined whether cranial muscle development was affected in *ring1b* mutants. To detect the developing muscles we used MF20, an antibody that recognizes the myosin heavy chain of vertebrate striated muscles [17].

The vertebrate craniofacial musculature is of paraxial mesoderm origin [4]. Muscle precursors residing in the first two pharyngeal arches give rise to muscles that are associated with the jaw and its support elements whereas precursors residing in arches III-VII form muscles associated with gill cartilage.

**Figure 2:**
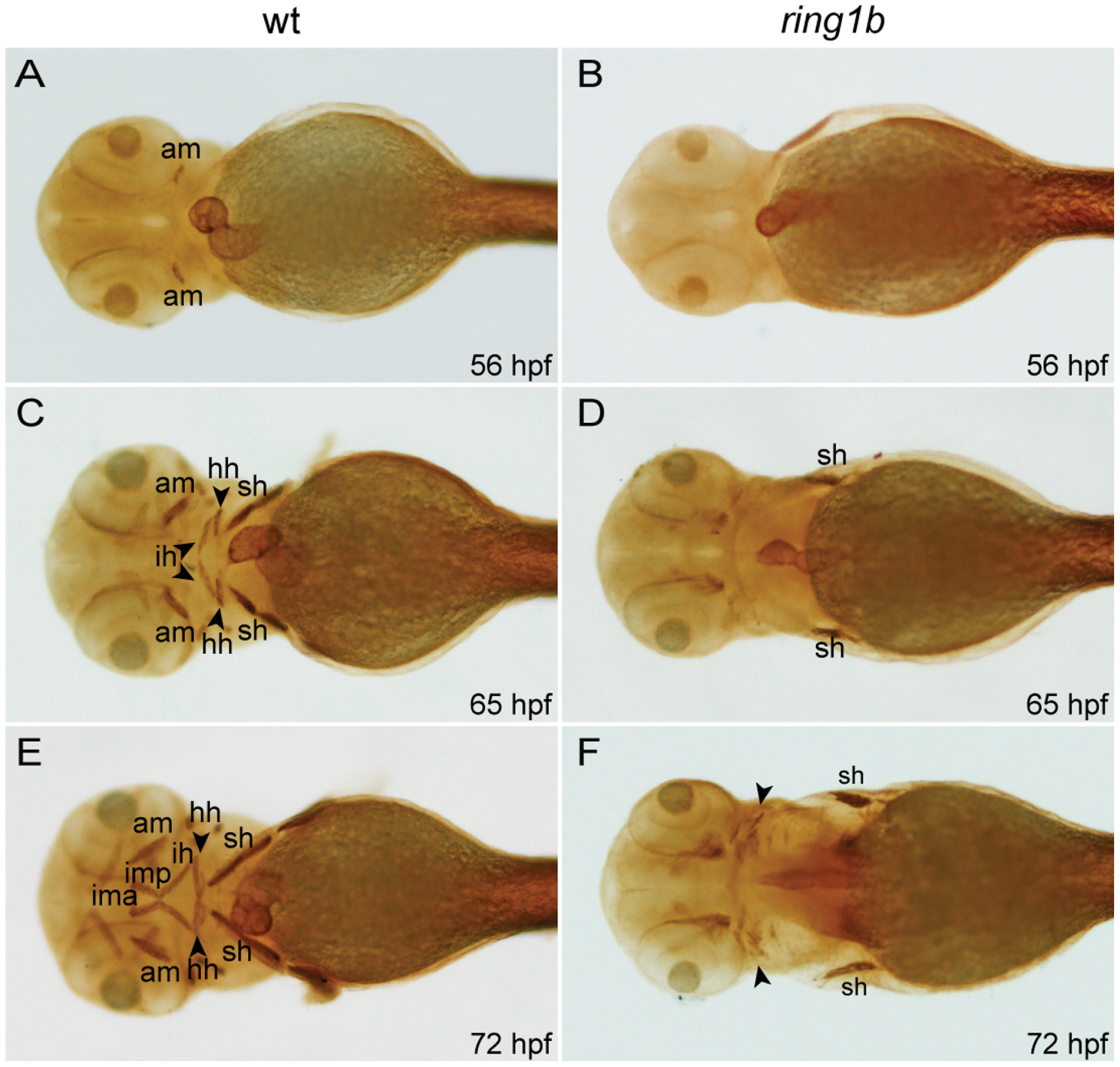
Cranial musculature development is severely impaired in *ring1b* mutants. Ventral views of WT and *ring1b* embryos stained with the MF20 antibody (A-F). The anterior mandibularis (am) has not formed in *ring1b* mutants at 56 hpf (B), the sternohyoideus (sh) is reduced at 65 hpf (D) and at 72 hpf, cranial muscles are almost completely absent (F). hh: hyohyoideus; ih: interhyoideus; ima: intermandibularis anterioris; imp: intermandibularis posterioris.

The anterior mandibularis that originates from the mandibular arch had formed by 56 hpf in WT embryos (Fig. 2A). At 65 hpf, cranial musculature associated with the jaw started to emerge (Fig. 2C). The hyohyoideus and interhyoideus, derived from the hyoid arch, matured further at 72 hpf and various other muscles, including the mandibular arch-derived intermandibularis anterioris, intermandibularis posterioris, levator arcus palatine and dilator operculi as well as the hyoid arch-derived adductor hyomandibulae and adductor opercule, were readily visible in WT embryos (Fig. 2E). In contrast, most craniofacial muscles were missing in *ring1b* mutants. Although *ring1b* mutants lack pectoral fins, a few MF20-positive cells, presumably pectoral fin muscle progenitors, were detected in-between the sternohyoideus and posterior hypaxial muscle (Fig. 2D). Only some irregular MF20-positive patches of craniofacial musculature were detected in 72 hpf *ring1b* mutants (Fig. 2F). We also analyzed development of somite-derived muscles and found that whereas initial formation and migration of those was unaffected, later elongation and differentiation was impaired in *ring1b* mutants (Fig. S1).

Our findings indicate that loss of Ring1b severely impairs the formation of craniofacial musculature, whereas initial formation and migration of somite-derived muscles is unaffected. This dramatic absence of cranial neural crest derived-tissues and head muscles in *ring1b* mutants prompted us to investigate whether all neural crest differentiation was affected.

### Development of Trunk Neural Crest Derivatives is Largely Unaffected in *ring1b* Mutants

NC cells give rise to a wide array of cell lineages including cartilage, bone, neurons, glia, smooth muscle cells and pigment cells. Since the differentiation of CNC cells into cartilage was abolished in *ring1b* mutants, we addressed whether overall NC differentiation, is perturbed by loss of Ring1B.

**Figure 3:**
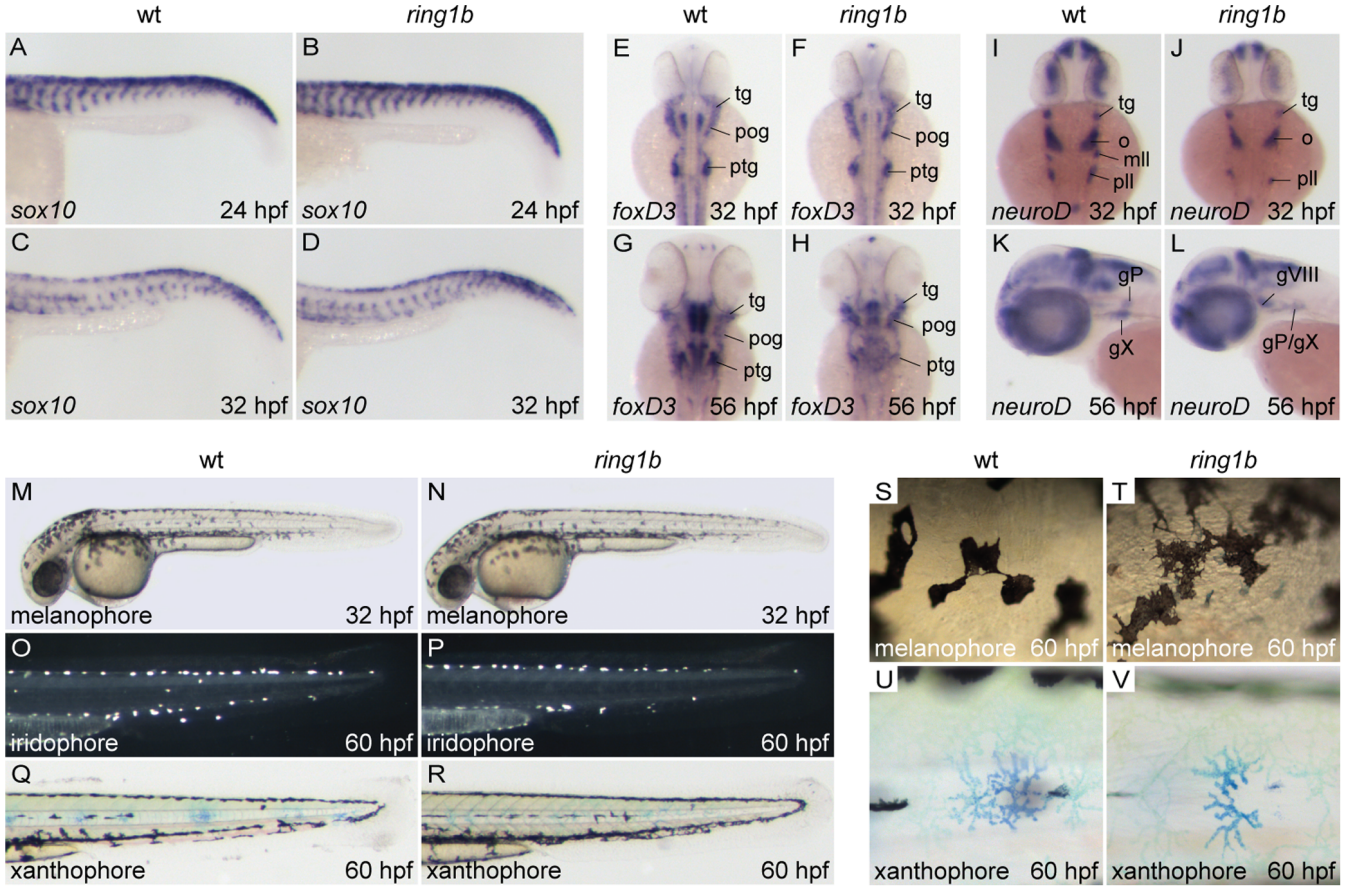
Migration and initial differentiation of trunk neural crest is largely unaffected in *ring1b* mutants. *Sox10* staining of WT and *ring1b* mutants at 24 and 32 hpf shows that *sox10* expressing NC cells have migrated timely and correctly to their ventral positions (A–D). Expression of *foxD3* in cranial ganglia-associated glia is comparable to WT siblings in *ring1b* mutants at 32 hpf (E, F), but reduced in the postotic ganglia of *ring1b* mutants at 56 hpf (G, H). *NeuroD* expression in cranial ganglia precursors is reduced in *ring1b* mutants at 32 hpf (I, J), but remains detectable in the hindbrain region of *ring1b* mutants at 56 hpf (L). Lateral view of 32 hpf embryos shows normal distribution of melanocytes in *ring1b* mutants at 32 hpf (M, N). At 60 hpf, *ring1b* melanophores remain more stellate than WT melanophores, which have started to round up (S, T). Iridophores are present in near normal numbers in *ring1b* mutants at 60 hpf (O, P), whereas *ring1b* xanthophores are smaller and less stellate (Q, R, U, V). Abbreviations: gX: vagal ganglia; mll: medial lateral line ganglia; o/gVIII: octaval/statoacustic ganglia; pll/gP: posteriolateral line ganglia; pog: preotic ganglia; ptg: postotic ganglia; tg: trigeminal ganglia.

To examine whether migration of trunk NC cells was affected, we stained embryos for *sox10,* a marker of NC lineages that is involved in the specification of non-ectomesenchymal NC derivatives, such as chromatophores, neurons and glia [18]. In WT embryos at 24 hpf, *Sox10*-positive NC cells were observed migrating ventrally from their dorsal pre-migratory position in a rostrocaudal fashion (Fig. 3A). At 24 hpf, we observed that *ring1b* trunk NC cells had migrated as far as in WT siblings and the localization of *sox10*-expressing NC cells remained indistinguishable from WT siblings at 32 hpf (Fig. 3A–D). Thus, the migration of trunk NC cells and their specification is normal in *ring1b* mutants.

To address whether glial differentiation was hindered, temporal expression of the forkhead transcription factor *foxD3* [19] was evaluated. In 32 hpf *ring1b* mutants, *foxD3* was expressed at near-normal levels in the cranial ganglia-associated glia of the developing trigeminal ganglion as well as in the preotic and postotic ganglia (Fig. 3E, F). At 56 hpf, *foxD3* was present in all domains of expression in *ring1b* mutants, albeit slightly weaker when compared to WT siblings (Fig. 3H). These data show that migration and initial differentiation of cranial glial precursors was largely normal. Next, we analyzed the expression of *neuroD*, a marker for all neurogenic placodes [20]. At 32 hpf, *neuroD* was expressed in the trigeminal ganglia, octaval/statoacustic ganglia, medial lateral line ganglia and the posteriolateral line ganglia in WT embryos (Fig. 3I). In *ring1b* mutants, *neuroD* expression was present but weaker than in WT (Fig. 3J). *Ring1b* mutants at 56 hpf were expressing *neuroD* in the octaval/statoacustic ganglia (gVIII) as well as in a single domain in which the vagal nerve (gX) and the posteriolateral line ganglia (gP) are located (Fig. 3K, L), which together showed that cranial ganglia development is largely unaffected.

Trunk neural crest gives rise also to pigment cells and therefore we morphologically inspected chromatophore development. Three NC-derived chromatophores are produced in zebrafish: melanophores (black), iridophores (iridescent) and xanthophores (yellow). While melanophores are found in a wide variety of vertebrates, iridophores and xanthophores are not [21]. At 32 hpf, melanophore pigmentation in *ring1b* mutants was indistinguishable from WT siblings. (Fig. 3M, N) During later stages of development, melanophores in WT embryos matured further and changed their morphology from spindly to a more compacted shape (Fig. 3S). In contrast, *ring1b* melanophores remained spindly (Fig. 3T). It has been reported however, that melanophores in 72 hpf WT embryos from a different genetic background displayed a very similar morphology to those in 60 hpf *ring1b* mutants [22]. Iridophores terminally differentiate at around 42 hpf and initially populate the dorsal and ventral stripe [23]. At 60 hpf, iridophores were present in both WT and *ring1b* mutants, albeit the number of iridophores was slightly reduced in the mutants (Fig. 3Q, R, U, V). Yellow xanthophores also emerged at around 42 hpf [24]. Since individual xanthophores are difficult to distinguish, qualitative observations can be made by staining for methylene blue, which is specifically taken up by xanthophores [25]. Methylene blue staining showed that xanthophores were present in the tail of both WT and *ring1b* mutants (Fig. 3Q, R). In addition, while melanocytes in WT embryos formed compacted islands, they appeared more ‘diffuse’ in the *ring1b* mutants (Fig. 3T) although that could be due to genetic background differences. Xanthophores visualized with methylene blue were present but displayed a different shape in the *ring1b* mutants (Fig. 3V).

Together, these results allow us to conclude that migration and differentiation of non-ectomesenchymal NC derivatives is only modestly affected by the loss of Ring1b. Glia, neurons and the three chromatophore lineages were formed in *ring1b* mutants. Thus, the arrest in cartilage formation appears to be a specific developmental defect in *ring1b* CNC cells.

### Ring1b is not Required for Early CNC Specification and Migration

Because craniofacial cartilage development was impaired in *ring1b* mutants, we investigated whether CNC cells that will form the prospective viscerocranium migrated correctly into the pharyngeal arches.

**Figure 4:**
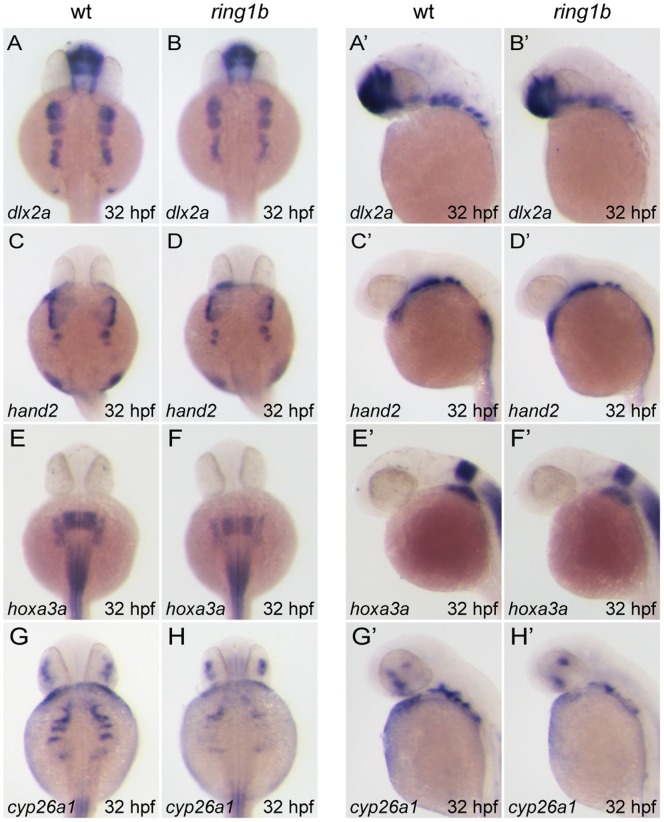
Cranial neural crest cells migrate into the pharyngeal arches of *ring1b* mutants. Dorsal (A–H) and lateral (A’–H’) views of WT and *ring1b* mutants at 32 hpf stained with the indicated genes. Pre-chondrogenic gene expression in CNCs is largely unaffected in *ring1b* mutants. *Dlx2a* is expressed at normal levels in the pharyngeal arches of *ring1b* mutants (A–B’). Separation of *dlx2a*-positive CNCs into distinct groups of cells that populate the posterior arches is slightly affected in *ring1b* mutants A–B’). *Hand2* expression in the *ring1b* pharyngeal arches is identical to that of WT siblings (C–D’). *Hoxa3a* expression is reduced in the *ring1b* posterior pharyngeal arches (E–F’). Expression of the pharyngeal pouch marker *cyp26a1* is severely reduced in *ring1b* mutants (G–H’).

We analyzed the expression of two transcription factors that are required for pharyngeal arch development, *dlx2a* that is expressed in CNC cells and *hand2*, whose expression is confined to a ventral subset of *dlx2a*-positive cells in post-migratory CNC cells [26], [27]. We observed that at 32 hpf, *dlx2a*-expressing CNC cells had migrated into the pharyngeal arches in both WT and *ring1b* embryos (Fig. 4A, B). In WT embryos, CNC cells had begun to separate into five cell groups that populate the branchial arches: three distinct cell groups were readily visible. The three branchial *dlx2a*-expressing cell groups were also detected in *ring1b* mutants but their separation appeared slightly affected. *Hand2* expression in the pharyngeal, hyoid and the two anterior branchial arches of *ring1b* mutants was indistinguishable from WT embryos (Fig. 4C, D). We also analyzed the expression of *hoxa3a*, a reported direct target of PcG proteins in mammals [28] that is expressed in the branchial arches (Fig. 4E, F) [29]. *Hoxa3a* expression was slightly reduced in *ring1b* mutants, but the overall size of the expression domain was unaffected.

Branchial pouches are outpockets of the foregut endoderm that play a role in directing CNC cell migration [15] and express *cyp26a1*. Expression of *cyp26a1* was reduced in *ring1b* mutants (Fig. 4G, H), indicating that pharyngeal pouch development was impaired.

Together, these data show that Ring1b is dispensable for the specification and migration of CNC cells into the pharyngeal arches. In addition, pharyngeal pouch development is affected, as assessed by reduced *cyp26a1* expression, which might contribute to the defect in cartilage development.

### Ring1b is Required for Chondrocyte Differentiation and Ossification

**Figure 5:**
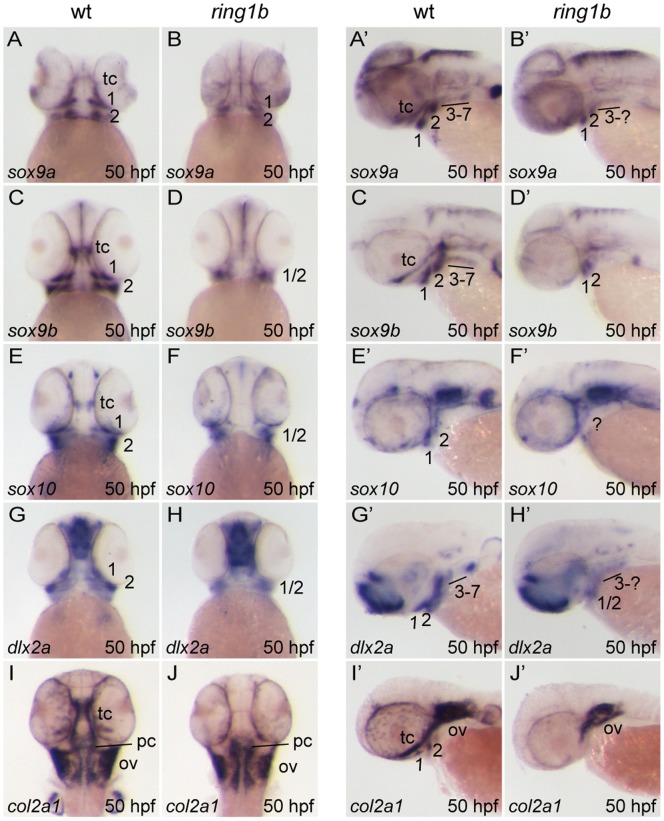
Impaired formation of prechondrogenic condensations and abolished cartilage differentiation in *ring1b* mutants. Ventral (A–J) and lateral (A’–J’) views of WT and *ring1b* mutants at 50 hpf stained with the indicated genes. Prechondrogenic condensation markers *sox9a* and *sox9b* are expressed in *ring1b* CNCs residing in pharyngeal arches 1 and 2, albeit the expression domain is smaller (A–D’). Similarly, the expression domain of CNC markers *sox10* and *dlx2a* is reduced in *ring1b* pharyngeal arches 1 and 2 (E–H’). Expression of s*ox9a, sox9b* and *sox10* is not detected at the presumptive location of the trabeculae in *ring1b* mutants (B, B’, D, D’, F, F’). *Col2a1* is expressed in WT trabeculae and pharyngeal arches I–II, but expression is abolished in *ring1b* mutants (I–, J’). Note that expression of *col2a1*, a marker for differentiating chondrocytes, is normal in the otic vesicles and parachordals of *ring1b* mutants. tc: trabeculae cranii; pc: parachordal; ov: otic vesicle. Numbers indicate the respective pharyngeal arches.

We next investigated whether differentiation of post-migratory CNC cells into chondrocytes was impaired. We stained WT and *ring1b* embryos with markers revealing differentiating cartilage. The transcription factors *sox9a/sox9b* have a critical role in cartilage morphogenesis [30], [31]. In 50 hpf WT embryos, *sox9a* and *sox9b* were expressed in prechondrogenic condensations of the mandibular arch (1), hyoid arch (2) and the posterior branchial arches (3–7) (Fig. 5A, A’, B, B’). In addition, both genes were expressed in the developing trabeculae cranii. In *ring1b* mutants, *sox9a* was expressed in the pharyngeal arches (Fig. 5B, B’), albeit the domain of expression was smaller. Interestingly, *ring1b* mutants lacked trabecular staining. *Sox9b* was expressed similarly to *sox9a* in the pharyngeal arches of WT embryos, whereas the trabecular expression domain was more extended (Fig. 5C, C’). *Sox9b* was expressed at reduced levels in arches 1,2 in *ring1b* mutants, whereas expression in arches 3–7 and at the presumptive trabeculae was missing (Fig. 5D, D’). Notably, *sox9a* and *sox9b* expression in the hindbrain domains was unaffected in the mutants, suggesting a specific defect in cartilage differentiation (and not a general downregulation of *sox9a/b* gene expression).

Similar results were obtained when examining the pan-neural crest markers *sox10* and *dlx2a* that remained expressed in post-migratory CNC cells. Both genes were expressed in the two anterior pharyngeal arches of *ring1b* mutants, whereas trabecular expression was completely absent (Fig. 5E–H, E’–H’).

To further examine chondrogenesis in *ring1b* mutants, we analyzed the expression of alpha I chain of type II collagen (*col2a1*), the major collagen in cartilage and marker of differentiating chondrocytes in zebrafish [32]. *Col2a1* expression is controlled by *sox9a/b* [31]. In 50 hpf WT embryos, *col2a1* was strongly expressed in the trabeculae cranii, otic vesicle and to a lesser extent in the mandibular and hyoid arch (Fig. 5I, I’). *Col2a1* was not expressed at the location of the presumptive trabeculae cranii in *ring1b* mutants (Fig. 5J, J’). Interestingly, *col2a1* was not expressed in *ring1b* mandibular and hyoid arches, despite the observed *sox9a/b* expression in these structures.

Overall, these results demonstrate that Ring1b is required for proper execution of the chondrocyte differentiation program.

**Figure 6:**
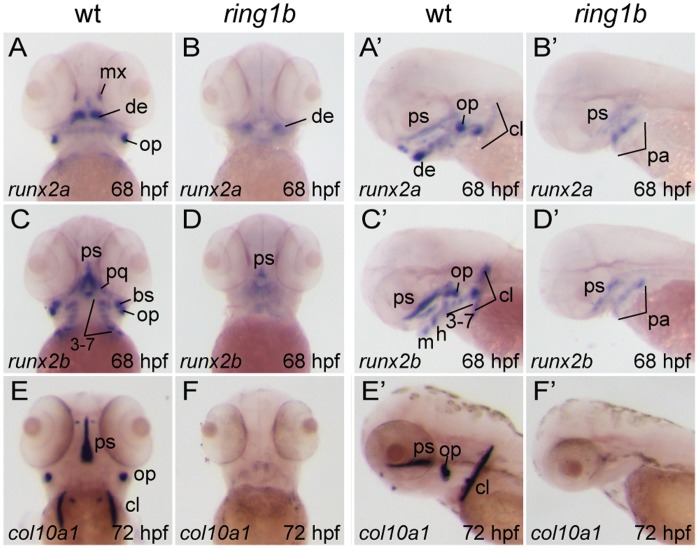
Loss of endochondral and dermal ossification in *ring1b* mutants. Ventral (A–D, E, F) and lateral (A’–D’, E’,) views of *in situ* hybridizations with riboprobes against the indicated genes in WT and *ring1b* mutants at 68–72 hpf. In WT embryos, *runx2a* and *runx2b* are expressed in hypertrophic pharyngeal arch-derived chondrocytes, as well as in the dermal ossification centers of the operculum, parasphenoid and cleithrum. Weak expression is detected in pharyngeal arches and the parasphenoid of *ring1b* mutants (B, B’, D, D’). At 72 hpf, *col10a1* is expressed in developing dermal bones in WT embryos, but not in *ring1b* mutants (E–F). cl: cleithrum; de: dentary; h: hyoid; m: mandibular; mx: maxilla; pa: pharyngeal arches; pq: palatoquadrate; ps: parasphenoid, op: operculum cl: the cleithrum. Numbers indicate the respective pharyngeal arches.

To address to which extent ossification is affected in *ring1b* mutants, we assayed for expression of the two *runx2* genes that are present in zebrafish, *runx2a* and *runx2b*. The transcription factor *runx2* is a key regulator of osteoblast differentiation and is also critically involved in the maturation step from immature chondrocytes to hypertrophic chondrocytes during the process of endochondral ossification [33], [34], [35]. In WT embryos, *runx2a* and *runx2b* were expressed in hypertrophic chondrocytes and dermal ossification centers. *Runx2a* was expressed in the cleithrum, dentary, maxilla, operculum, pharyngeal arches and parasphenoid whereas *runx2b* expression was detected in differentiating osteoblasts of the branchiostegial ray, cleithrum, operculum, palatoquadrate, parasphenoid and pharyngeal arches (Fig. 6A, C).

*Runx2a* and *runx2b* expression was greatly reduced or absent in presumptive cartilaginous elements of the viscerocranium in 68 hpf *ring1b* mutants (Fig. 6B, D) indicating that endochondral ossification is abolished.

In addition, weak *runx2a/b* expression was detected only at the presumptive parasphenoid, suggesting that dermal ossification may also be disrupted. To confirm this observation, we stained for type X collagen (*col10a1*), an extracellular matrix component known to be directly regulated by *runx2* [36]. *Col10a1* expression is specific to developing dermal bones at 72 hpf. Indeed, strong staining was observed in the cleithrum, operculum and parasphenoid of WT embryos (Fig. 6E). Strikingly, *col10a1* expression was completely absent in *ring1b* mutants (Fig. 6F). These results show that dermal ossification is also severely impaired in *ring1b* mutants.

**Figure 7:**
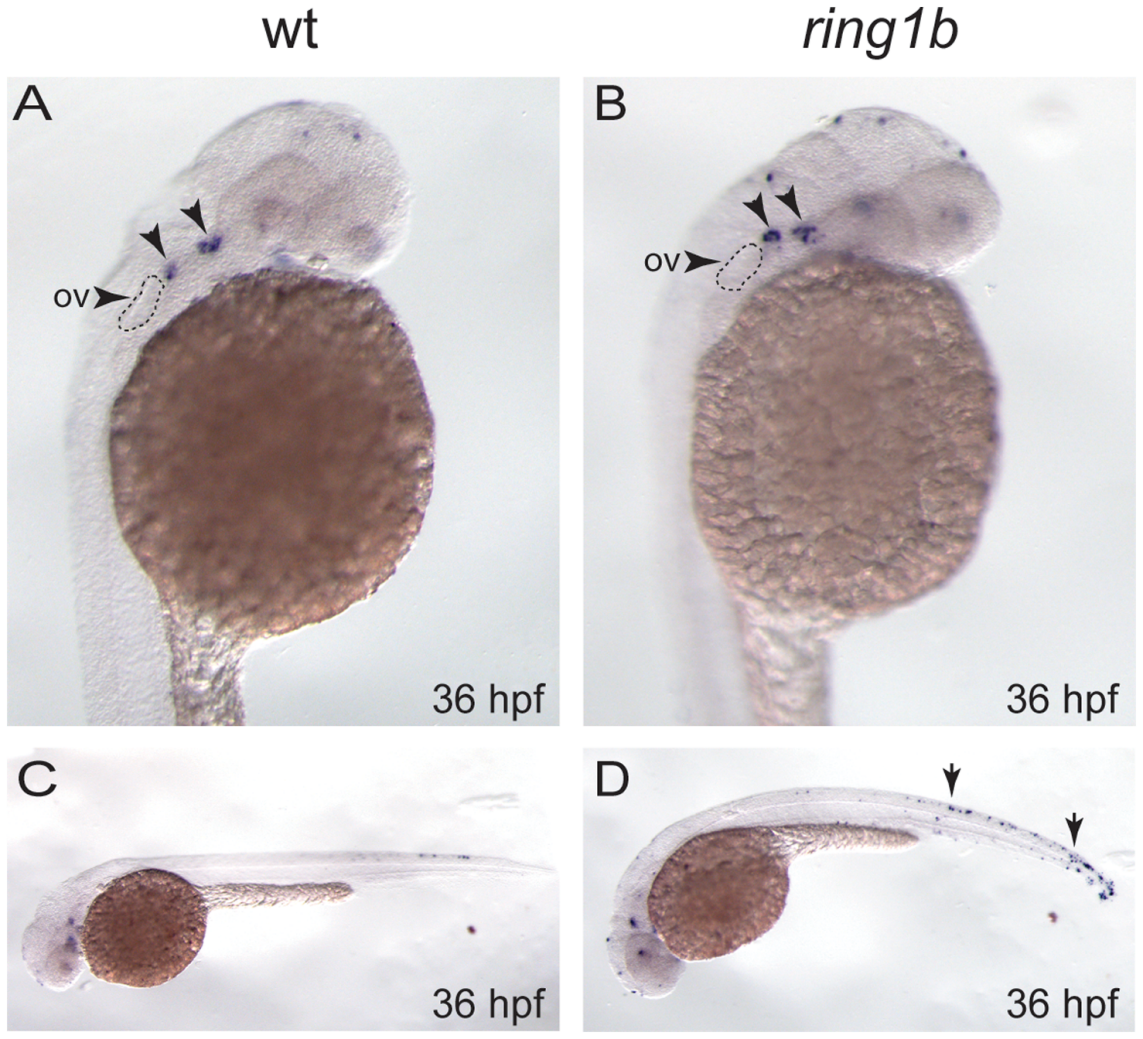
Apoptosis is slightly increased in the pharyngeal arch of *ring1b* mutants. Lateral views of WT and *ring1b* embryos at 36 hpf stained for TUNEL. In WT embryos two small clusters of TUNEL-positive apoptotic cells were detected in the pharyngeal arch region just posterior to the eye (A, arrows). These clusters appear to contain more apoptotic cells in the *ring1b* mutants (B, arrows). The arrowhead indicates the otic vesicle (ov). WT embryos at 36 hpf contain few apoptotic cells in the trunk (C), whereas there is an increase in overall apoptosis particularly in the trunk and the tail in *ring1b* mutants (D, arrows).

The marked reduction in the expression of genes involved in chondrocyte differentiation prompted us to investigate whether the cartilage progenitors could have been eliminated by apoptosis. To this end, we stained WT and *ring1b* embryos for apoptotic cells using TUNEL and Acridine Orange staining. We stained embryos at 36 hpf, since at this developmental stage the *ring1b* CNC cells express markers at similar levels as the WTs. As shown in Fig. 7A, WT embryos displayed two small clusters of TUNEL-positive apoptotic cells in the pharyngeal arch area and although in *ring1b* embryos the same clusters appeared to contain more apoptotic cells (Fig. 7B), the induction of apoptosis observed in this particular region was not considered significant. Nonetheless, *ring1b* embryos exhibited a robust overall increase in apoptosis when compared to WT siblings, particularly in the trunk and tail (Fig. 7C, D). Acridine orange staining was conducted in WT and *ring1b* embryos at 48 hpf when deregulations of gene expression were apparent in the mutants. Consistently with the enhanced cell death detected in *ring1b* embryos, we observed that a small number of apoptotic cells persisted at 48 hpf in the prospective jaw region, whereas no apoptotic cells could be detected in the WTs at this stage (Fig. S2A, B). Overall, these results indicate that the slight upregulation of apoptosis occurring in *ring1b* mutants may contribute to the elimination of few chondrocyte progenitors, however, it may not be sufficient to account for the almost complete lack of cartilage observed in *ring1b* mutants.

We show here that zebrafish deficient in the Polycomb gene *ring1b*/*rnf2* display severe craniofacial defects and lack cartilage elements of the neurocranium, pharyngeal arches, and pectoral girdle.

Craniofacial birth abnormalities result from defects in cranial neural crest patterning and morphogenesis. A better understanding of the molecular mechanisms that regulate chondrogenesis will facilitate development of therapies and/or prevention. The rapid development of the jaw cartilages in the zebrafish, its transparency and amenability to genetic manipulations makes it an excellent model for studying the genetic basis of craniofacial development. In addition, zebrafish mutants isolated in forward and reverse genetic screens have enabled the identification of critical genes involved in cartilage development [37], [38], [39]. Interestingly, several of those serve as models for human cartilage diseases, such as campomelic dysplasia (*sox9*) [39].

The dramatic *ring1b* cartilage phenotype is quite rare as only few mutants showing a total lack of pharyngeal elements and neurocranium have been identified. Remarkably, the *ring1b* mutant is the only one among those with cartilage defects that is missing the cleithrum, the pectoral girdle element that is not of Neural Crest origin. Not surprisingly, the *sox9a/sox9b* double mutant displayed severe craniofacial abnormalities. Zebrafish mutants in Trap230/Med12, a transcriptional co-activator for Sox9, were characterized by disrupted cartilage development [40], [41]. Furthermore, knockdown experiments showed that *runx2b* and *runx3* are critically involved in chondrogenesis: *runx2b* morphants lack the entire pharyngeal skeleton whereas only rudimentary trabeculae are formed in *runx3* morphants [42].

Finally, in the *lim-absent* (*lia*)/*fgf3* mutant, posterior arch formation is abrogated [43]. Potent inhibition of Fgf signaling, either chemically or by morholino-mediated knockdown of both Fgf3 and Fgf8, led to an almost complete loss of cartilage [44].

Our data indicate that Ring1b acts genetically upstream of Sox9 and Runx2, as their expression in (pre)chondrocytes is markedly decreased in *ring1b* mutants. We demonstrated previously that the Fgf-target genes *dusp6*, *pea3* and *spry4* were expressed at near normal levels in the pharyngeal arches of 32 hpf *ring1b* mutants [13], indicating that impaired Fgf signaling is unlikely to be underlying the cartilage defect.

Chondrogenesis involves a cascade of events that requires the concerted action of an intricate gene regulatory network. Although recent studies have provided insight into the molecular mechanisms underlying chondrogenesis, much remains unknown. For instance, the role of (epigenetic) chromatin modifications such as chromatin remodeling, DNA (de) methylation and histone modifications has largely been overlooked. Our results illustrate the critical role of epigenetic control in cartilage differentiation and highlight for the first time an essential and specific role for Polycomb genes in craniofacial development.

Despite the numerous molecular targets of Polycomb-mediated repression, the *ring1b* cartilage phenotype affects specific groups of cells and at a specific developmental point, namely the late stages of differentiation of the cranial neural crest cells into cartilage and muscle of the head. Our results indicate that epigenetic regulation of transcription in specific cell populations is very important for late differentiation events.

The cartilage phenotype in *ring1b* mutants consists of a block in terminal differentiation of chondrocytes. We propose that this is the result of deregulated expression of developmental programs rather than individual genes. This is in line with the proposed role of Ring1b to control differentiation programs in ES cells and of the Polycomb complex to regulate programs rather than individual genes.

Since no cartilage is formed in *ring1b* mutants, they provide an attractive platform to interrogate the function of (human) candidate genes for skeletogenesis, dissect possible genetic interactions, as well as screening of compounds to identify regulators of cartilage development.

## Materials and Methods

### Zebrafish Strains and Genotyping Methods

Zebrafish (*Danio rerio*) were maintained as previously described [45]. They were cared for in accordance with the NKI institutional guidelines and as approved by the Animal Experimentation Committee (DEC) of the Royal Netherlands Academy of Arts and Sciences (KNAW). *Ring1b* founder fish were out-crossed to AB and TL genetic backgrounds and genotyped as described [13] The mutation in *ring1b*/*rnf2* that led to the insertion of 4 base pairs (bp) within the ZFN target site was named *rnf2 ^ibl30/ibl30^* and the mutation that caused deletion of 14 bp at the ZFN target site was named *rnf2 ^ibl31/ibl31^* (www.zfin.org).

### Whole-mount *in situ* Hybridization, Antibody and Cartilage Staining

Whole-mount *in situ* hybridizations were carried out according to standard protocols [45]. Probes for *dlx2a*, *col2a1*, *col10a*, *hand2*, *runx2a*, *runx2b*, *sox9a* and *sox9b* were previously described. For *cyp26a1*, *foxD3*, *hoxa3a*, *neuroD*, and *sox10,* antisense riboprobes were amplified from cDNA. Primer sequences are available upon request.

## Supporting Information

Figure S1 **Cranial musculature development is severely impaired in** ***ring1b*** **mutants.** Lateral views of embryonic musculature in WT and *ring1b* mutants (A–H). The posterior hypaxial muscle (phm), had delaminated from the somites in both WT and *ring1b* mutants at 48 hpf (A, B). At 56 hpf, the sternohyoideus (sh) is formed in both WT and *ring1b* mutants and the pectoral fin muscle is prominently visible (C, D). During later development, the sh and phm elongated and attached to the cleithrum, a bone of the fin girdle in WT embryos, (E, G). Elongation of these muscles was completely abrogated in *ring1b* mutants (F, H). Cranial musculature is almost completely absent in *ring1b* mutants (H). Abbreviations: am: anterior mandibularis; ah: adductor hyomandibulae; ao: adductor opercule; do: dilator operculi; hh: hyohyoideus; ih: interhyoideus; ima: intermandibularis anterioris; imp: intermandibularis posterioris, lap: levator arcus palatine; phm: posterior hypaxial muscle; pfm: pectoral fin muscle; sh: sternohyoideus.
(TIF)

Figure S2 **Persistence of apoptotic cells in** ***ring1b*** **mutants.** Ventral views of WT and *ring1b* embryos at 48 hpf stained with Acridine Orange. No apoptotic cells are detected in the pharyngeal arch region of WT embryos (A) whereas few AO-positive apoptotic cells have persisted in the *ring1b* mutants (B) Arrows in (B) indicate apoptotic clusters in the prospective jaw region and anteriorly to the otic vesicle. Figure also shows images of live WT and *ring1b* embryos depicting the fin and pharyngeal cartilages in WT (C, arrows) and their absence in the *ring1b* mutants (D, *).
(TIF)

Methods S1 (DOCX)

## References

[1] Gans C, Northcutt RG (1983) Neural crest and the origin of vertebrates: a new head. Science 220: 268–273. 17732898 10.1126/science.220.4594.268

[2] LaBonne C, Bronner-Fraser M (1999) Molecular mechanisms of neural crest formation. Annu Rev Cell Dev Biol 15: 81–112. 10611958 10.1146/annurev.cellbio.15.1.81

[3] Wada N, Javidan Y, Nelson S, Carney TJ, Kelsh RN, et al (2005) Hedgehog signaling is required for cranial neural crest morphogenesis and chondrogenesis at the midline in the zebrafish skull. Development 132: 3977–3988. 16049113 10.1242/dev.01943

[4] Schilling TF, Kimmel CB (1994) Segment and cell type lineage restrictions during pharyngeal arch development in the zebrafish embryo. Development 120: 483–494. 8162849 10.1242/dev.120.3.483

[5] Raible DW, Eisen JS (1994) Restriction of neural crest cell fate in the trunk of the embryonic zebrafish. Development 120: 495–503. 8162850 10.1242/dev.120.3.495

[6] Sparmann A, van Lohuizen M (2006) Polycomb silencers control cell fate, development and cancer. Nat Rev Cancer 6: 846–856. 17060944 10.1038/nrc1991

[7] Simon JA, Kingston RE (2009) Mechanisms of polycomb gene silencing: knowns and unknowns. Nature reviews Molecular cell biology 10: 697–708. 19738629 10.1038/nrm2763

[8] Simon JA, Kingston RE (2013) Occupying chromatin: polycomb mechanisms for getting to genomic targets, stopping transcriptional traffic, and staying put. Molecular cell 49: 808–824. 23473600 10.1016/j.molcel.2013.02.013 PMC3628831

[9] Eskeland R, Leeb M, Grimes GR, Kress C, Boyle S, et al (2010) Ring1B compacts chromatin structure and represses gene expression independent of histone ubiquitination. Mol Cell 38: 452–464. 20471950 10.1016/j.molcel.2010.02.032 PMC3132514

[10] de Napoles M, Mermoud JE, Wakao R, Tang YA, Endoh M, et al (2004) Polycomb group proteins Ring1A/B link ubiquitylation of histone H2A to heritable gene silencing and X inactivation. Dev Cell 7: 663–676. 15525528 10.1016/j.devcel.2004.10.005

[11] Wang H, Wang L, Erdjument-Bromage H, Vidal M, Tempst P, et al (2004) Role of histone H2A ubiquitination in Polycomb silencing. Nature 431: 873–878. 15386022 10.1038/nature02985

[12] Voncken JW, Roelen BA, Roefs M, de Vries S, Verhoeven E, et al (2003) Rnf2 (Ring1b) deficiency causes gastrulation arrest and cell cycle inhibition. Proc Natl Acad Sci U S A 100: 2468–2473. 12589020 10.1073/pnas.0434312100 PMC151364

[13] van der Velden YU, Wang L, van Lohuizen M, Haramis AP The Polycomb group protein Ring1b is essential for pectoral fin development. Development 139: 2210–2220. 10.1242/dev.077156 22619390

[14] Meng X, Noyes MB, Zhu LJ, Lawson ND, Wolfe SA (2008) Targeted gene inactivation in zebrafish using engineered zinc-finger nucleases. Nat Biotechnol 26: 695–701. 18500337 10.1038/nbt1398 PMC2502069

[15] Knight RD, Schilling TF (2006) Cranial neural crest and development of the head skeleton. Adv Exp Med Biol 589: 120–133. 17076278 10.1007/978-0-387-46954-6_7

[16] Schilling TF, Kimmel CB (1997) Musculoskeletal patterning in the pharyngeal segments of the zebrafish embryo. Development 124: 2945–2960. 9247337 10.1242/dev.124.15.2945

[17] Bader D, Masaki T, Fischman DA (1982) Immunochemical analysis of myosin heavy chain during avian myogenesis in vivo and in vitro. J Cell Biol 95: 763–770. 6185504 10.1083/jcb.95.3.763 PMC2112936

[18] Dutton KA, Pauliny A, Lopes SS, Elworthy S, Carney TJ, et al (2001) Zebrafish colourless encodes sox10 and specifies non-ectomesenchymal neural crest fates. Development 128: 4113–4125. 11684650 10.1242/dev.128.21.4113

[19] Kelsh RN, Dutton K, Medlin J, Eisen JS (2000) Expression of zebrafish fkd6 in neural crest-derived glia. Mech Dev 93: 161–164. 10781949 10.1016/s0925-4773(00)00250-1

[20] Andermann P, Ungos J, Raible DW (2002) Neurogenin1 defines zebrafish cranial sensory ganglia precursors. Dev Biol 251: 45–58. 12413897 10.1006/dbio.2002.0820

[21] Curran K, Raible DW, Lister JA (2009) Foxd3 controls melanophore specification in the zebrafish neural crest by regulation of Mitf. Dev Biol 332: 408–417. 19527705 10.1016/j.ydbio.2009.06.010 PMC2716409

[22] Kelsh RN, Brand M, Jiang YJ, Heisenberg CP, Lin S, et al (1996) Zebrafish pigmentation mutations and the processes of neural crest development. Development 123: 369–389. 9007256 10.1242/dev.123.1.369

[23] Curran K, Lister JA, Kunkel GR, Prendergast A, Parichy DM, et al (2010) Interplay between Foxd3 and Mitf regulates cell fate plasticity in the zebrafish neural crest. Dev Biol 344: 107–118. 20460180 10.1016/j.ydbio.2010.04.023 PMC2909359

[24] Odenthal J, Rossnagel K, Haffter P, Kelsh RN, Vogelsang E, et al (1996) Mutations affecting xanthophore pigmentation in the zebrafish, Danio rerio. Development 123: 391–398. 9007257 10.1242/dev.123.1.391

[25] Le Guyader S, Jesuthasan S (2002) Analysis of xanthophore and pterinosome biogenesis in zebrafish using methylene blue and pteridine autofluorescence. Pigment Cell Res 15: 27–31. 11837453 10.1034/j.1600-0749.2002.00045.x

[26] Akimenko MA, Ekker M, Wegner J, Lin W, Westerfield M (1994) Combinatorial expression of three zebrafish genes related to distal-less: part of a homeobox gene code for the head. J Neurosci 14: 3475–3486. 7911517 10.1523/JNEUROSCI.14-06-03475.1994 PMC6576961

[27] Angelo S, Lohr J, Lee KH, Ticho BS, Breitbart RE, et al (2000) Conservation of sequence and expression of Xenopus and zebrafish dHAND during cardiac, branchial arch and lateral mesoderm development. Mech Dev 95: 231–237. 10906469 10.1016/s0925-4773(00)00334-8

[28] Bracken AP, Dietrich N, Pasini D, Hansen KH, Helin K (2006) Genome-wide mapping of Polycomb target genes unravels their roles in cell fate transitions. Genes Dev 20: 1123–1136. 16618801 10.1101/gad.381706 PMC1472472

[29] Hogan BM, Hunter MP, Oates AC, Crowhurst MO, Hall NE, et al (2004) Zebrafish gcm2 is required for gill filament budding from pharyngeal ectoderm. Dev Biol 276: 508–522. 15581882 10.1016/j.ydbio.2004.09.018

[30] Yan YL, Miller CT, Nissen RM, Singer A, Liu D, et al (2002) A zebrafish sox9 gene required for cartilage morphogenesis. Development 129: 5065–5079. 12397114 10.1242/dev.129.21.5065

[31] Yan YL, Willoughby J, Liu D, Crump JG, Wilson C, et al (2005) A pair of Sox: distinct and overlapping functions of zebrafish sox9 co-orthologs in craniofacial and pectoral fin development. Development 132: 1069–1083. 15689370 10.1242/dev.01674

[32] Yan YL, Hatta K, Riggleman B, Postlethwait JH (1995) Expression of a type II collagen gene in the zebrafish embryonic axis. Dev Dyn 203: 363–376. 8589433 10.1002/aja.1002030308

[33] Komori T, Yagi H, Nomura S, Yamaguchi A, Sasaki K, et al (1997) Targeted disruption of Cbfa1 results in a complete lack of bone formation owing to maturational arrest of osteoblasts. Cell 89: 755–764. 9182763 10.1016/s0092-8674(00)80258-5

[34] Otto F, Thornell AP, Crompton T, Denzel A, Gilmour KC, et al (1997) Cbfa1, a candidate gene for cleidocranial dysplasia syndrome, is essential for osteoblast differentiation and bone development. Cell 89: 765–771. 9182764 10.1016/s0092-8674(00)80259-7

[35] Nakashima K, Zhou X, Kunkel G, Zhang Z, Deng JM, et al (2002) The novel zinc finger-containing transcription factor osterix is required for osteoblast differentiation and bone formation. Cell 108: 17–29. 11792318 10.1016/s0092-8674(01)00622-5

[36] Li F, Lu Y, Ding M, Napierala D, Abbassi S, et al (2011) Runx2 contributes to murine Col10a1 gene regulation through direct interaction with its cis-enhancer. J Bone Miner Res 26: 2899–2910. 21887706 10.1002/jbmr.504 PMC3222790

[37] Neuhauss SC, Solnica-Krezel L, Schier AF, Zwartkruis F, Stemple DL, et al (1996) Mutations affecting craniofacial development in zebrafish. Development 123: 357–367. 9007255 10.1242/dev.123.1.357

[38] Schilling TF, Piotrowski T, Grandel H, Brand M, Heisenberg CP, et al (1996) Jaw and branchial arch mutants in zebrafish I: branchial arches. Development 123: 329–344. 9007253 10.1242/dev.123.1.329

[39] Nissen RM, Amsterdam A, Hopkins N (2006) A zebrafish screen for craniofacial mutants identifies wdr68 as a highly conserved gene required for endothelin-1 expression. BMC Dev Biol 6: 28. 16759393 10.1186/1471-213X-6-28 PMC1523201

[40] Rau MJ, Fischer S, Neumann CJ (2006) Zebrafish Trap230/Med12 is required as a coactivator for Sox9-dependent neural crest, cartilage and ear development. Dev Biol 296: 83–93. 16712834 10.1016/j.ydbio.2006.04.437

[41] Hong SK, Haldin CE, Lawson ND, Weinstein BM, Dawid IB, et al (2005) The zebrafish kohtalo/trap230 gene is required for the development of the brain, neural crest, and pronephric kidney. Proc Natl Acad Sci U S A 102: 18473–18478. 16344459 10.1073/pnas.0509457102 PMC1311743

[42] Flores MV, Lam EY, Crosier P, Crosier K (2006) A hierarchy of Runx transcription factors modulate the onset of chondrogenesis in craniofacial endochondral bones in zebrafish. Dev Dyn 235: 3166–3176. 17013873 10.1002/dvdy.20957

[43] Herzog W, Sonntag C, von der Hardt S, Roehl HH, Varga ZM, et al (2004) Fgf3 signaling from the ventral diencephalon is required for early specification and subsequent survival of the zebrafish adenohypophysis. Development 131: 3681–3692. 15229178 10.1242/dev.01235

[44] David NB, Saint-Etienne L, Tsang M, Schilling TF, Rosa FM (2002) Requirement for endoderm and FGF3 in ventral head skeleton formation. Development 129: 4457–4468. 12223404 10.1242/dev.129.19.4457

[45] Westerfield M (2000) The Zebrafish Book. Eugene: University of Oregon Press.

