# Supplementary figures and images for: The Polycomb Group Protein Ring1b/Rnf2 Is Specifically Required for Craniofacial Development
Source: PLoS One. 2013 Sep 11;8(9):e73997. doi: 10.1371/journal.pone.0073997 (PMC3770662; doi:10.1371/journal.pone.0073997)

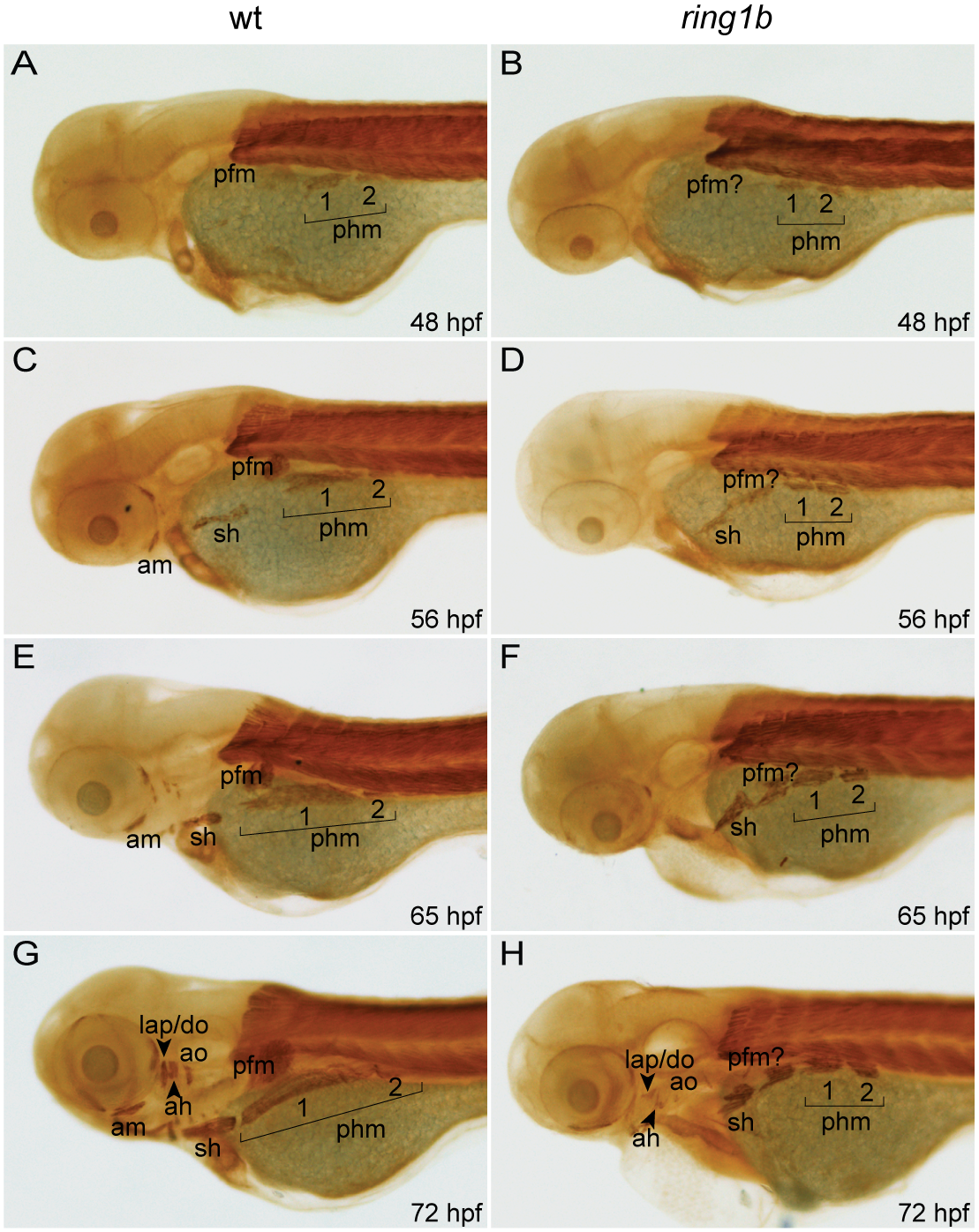

Supplement: Figure S1 — Cranial musculature development is severely impaired in ring1b mutants. Lateral views of embryonic musculature in WT and ring1b mutants (A–H). The posterior hypaxial muscle (phm), had delaminated from the somites in both WT and ring1b mutants at 48 hpf (A, B). At 56 hpf, the sternohyoideus (sh) is formed in both WT and ring1b mutants and the pectoral fin muscle is prominently visible (C, D). During later development, the sh and phm elongated and attached to the cleithrum, a bone of the fin girdle in WT embryos, (E, G). Elongation of these muscles was completely abrogated in ring1b mutants (F, H). Cranial musculature is almost completely absent in ring1b mutants (H). Abbreviations: am: anterior mandibularis; ah: adductor hyomandibulae; ao: adductor opercule; do: dilator operculi; hh: hyohyoideus; ih: interhyoideus; ima: intermandibularis anterioris; imp: intermandibularis posterioris, lap: levator arcus palatine; phm: posterior hypaxial muscle; pfm: pectoral fin muscle; sh: sternohyoideus. (TIF) [file pone.0073997.s001.tif]

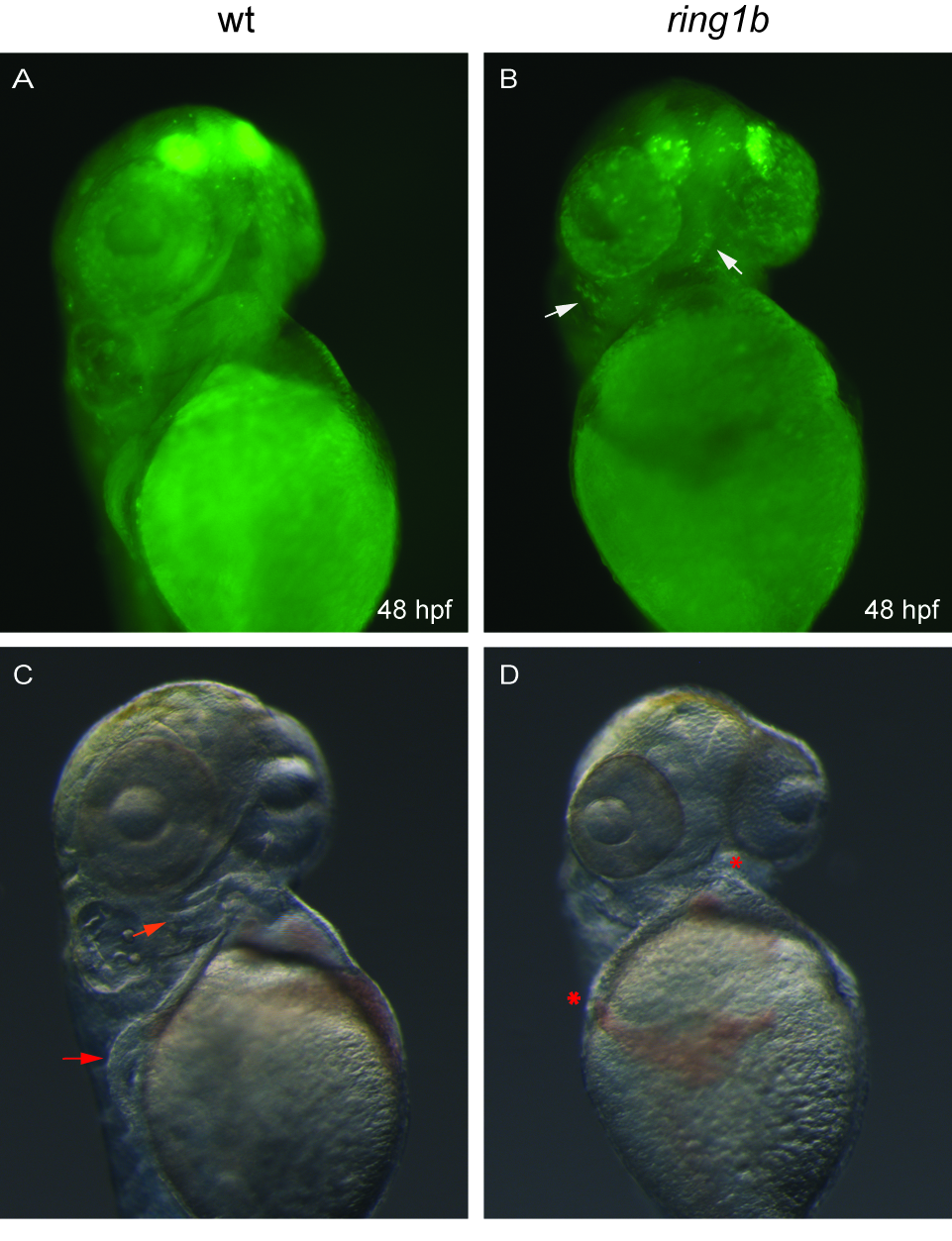

Supplement: Figure S2 — Persistence of apoptotic cells in ring1b mutants. Ventral views of WT and ring1b embryos at 48 hpf stained with Acridine Orange. No apoptotic cells are detected in the pharyngeal arch region of WT embryos (A) whereas few AO-positive apoptotic cells have persisted in the ring1b mutants (B) Arrows in (B) indicate apoptotic clusters in the prospective jaw region and anteriorly to the otic vesicle. Figure also shows images of live WT and ring1b embryos depicting the fin and pharyngeal cartilages in WT (C, arrows) and their absence in the ring1b mutants (D, *). (TIF) [file pone.0073997.s002.tif]
