# Supplementary material for: The Polycomb Group Protein Ring1b/Rnf2 Is Specifically Required for Craniofacial Development
Source: PLoS One. 2013 Sep 11;8(9):e73997. doi: 10.1371/journal.pone.0073997 (PMC3770662; doi:10.1371/journal.pone.0073997)
Supplement: Methods S1 — (DOCX) [file pone.0073997.s003.docx]

## Supplemental Materials and Methods

### Whole-mount cartilage staining

Embryos were fixed in 40% ethanol, 5% acetic acid, and 10% formalin containing 0.02% Alcian Blue for 6 hours at room temperature, followed by dehydration and overnight storage in 100% ethanol at -20 °C. Embryos were rehydrated and washed in MQ containing 0.2% Triton-X100. Pigment was bleached by 30 minute incubation in MQ containing 1% KOH, 3% H_2_O_2_ and 0.2% Triton-X100. After two washes in MQ containing 0.2% Triton-X100, the bleaching was neutralized by 10 minute incubation in a saturated Sodium tetraborate solution. Next, the embryos were digested in a 60% saturated tetraborate solution containing 0.01% trypsin (Sigma) for one hour. Embryos were cleared in a deionized water containing 20% glycerol, 1% KOH and 0.2% Triton-X100 for 20 minutes and stored in 70% glycerol.

### Whole-mount antibody staining

Embryos were fixed overnight in 20% DMSO, 80% Methanol, or 4% paraformaldehyde at 4 °C, followed by dehydration and overnight storage in methanol at -20°C. For MF-20 antibody staining, mouse anti-MF20 (Developmental Studies Hybridoma Bank, developed under the auspices of the NICHD and maintained by The University of Iowa, Department of Biology, Iowa City, IA 52242) was used at 1:20 dilution.

**Whole-mount TUNEL and Acridine Orange Staining**

Embryos were fixed overnight in 4% paraformaldehyde at 4 °C, followed by dehydration and storage in methanol at -20°C. Rehydrated embryos were digested with 10 μg/ml Proteinase K for 10 min at 37 °C, fixed in 4 % PFA for 20 min at room temperature (RT) and washed with TdT buffer (300 mM Tris/HCl; 1400 mM NaCacodylate) for 30 min prior to incubation with an enzyme mix containing Dig-dUTP, ATP and 100 U of TdT enzyme (Promega Benelux) for 4 h at 4 °C. 100 mM CoCl_2_ was added overnight to the solution. The following day, the reaction was stopped by adding 2mM EDTA for further 30 min at RT prior to washing in 10 % heat-inactivated sheep serum for 60 min at RT. Anti-Digoxigenin antibody (1:5000; Roche) was added and embryos were incubated overnight at 4 °C. Embryos were finally extensively washed in PBS prior washing in NTT buffer (0.1M Tris pH 9.5, 0.1M NaCl, 0.1% Tween20) and stained using BM Purple (Roche). Whole embryos were imaged by light transmission microscopy (Leica MZ16 FA) using the Leica software.

For Acridine Orange staining, live embryos were incubated for 30 minutes at 28 °C in egg water containing 5 μg/ml Acridine Orange (Chroma). Following extensive washes in egg water, embryos were anaesthetized in tricaine (Sigma) and imaged in a fluorescence stereomicroscope (Leica MZ16 FA) using the Leica software.
